# Editorial: PTCL from genes to therapy

**DOI:** 10.3389/fonc.2022.1014483

**Published:** 2022-09-16

**Authors:** Andrea Janikova

**Affiliations:** Department of Hematology and Oncology, University Hospital Brno, Brno, Czechia

**Keywords:** T-cell lymphoma, ALK (anaplastic lymphoma kinase), mycosis fungoides (MF), proteomics, lymphoma CNS

Mature T-cell-derived lymphomas (MTCLs) are rare hematological malignancies, which represent about 7%–10% of all newly diagnosed non-Hodgkin lymphomas (NHLs) only. Despite the rarity of this subgroup of malignant lymphomas, MTCLs are distinguished by marked molecular, clinical, and biologic heterogeneity and by prognosis much worse than in B-cell lymphomas. Research is urgently and continuously needed in this field. This special issue would like to contribute at least a little bit to a deeper understanding of malignant T-cell-derived diseases.

The epidemiology of T-cell lymphoma also exhibits a unique geographic diversity, with a high prevalence of T-cell and NK/T-cell lymphomas in Asian countries compared to the West. In Europe and the USA, the most frequent subtypes are anaplastic large cell lymphoma (ALCL; around 25% of cases), peripheral T-cell lymphoma not otherwise specified (PTCL-NOS; around 35% of cases), and angioimmunoblastic T-cell lymphoma (AITL; varying between 7% and 20% of cases). From a clinical point of view, T-cell lymphomas can be divided into several subgroups: nodal, extranodal, cutaneous, and leukemic. A subgroup of cutaneous T-cell lymphomas represents a heterogeneous collection of diseases that arise from skin-tropic memory T lymphocytes. Among them, mycosis fungoides (MF) and Sézary syndrome (SS) are the most common malignancies. The article *Retrospective analysis of 118 patients with cutaneous T-cell lymphomas: a single-center experience* written by Polgarova et al. brings the real-life results of middle European patients diagnosed between 1998 and 2021 treated with common non-targeted therapy (low-dose methotrexate, interferon-alpha, bexarotene, chlorambucil, extracorporeal photopheresis). This work is a retrospective descriptive analysis, which can also serve as a comparative basis for new therapeutic interventions in T-cell lymphoma of the skin. A specific subgroup of extranodal lymphomas is represented by primary or secondary central nervous system (CNS) lymphomas. Unlike diffuse large B-cell lymphoma of the brain, very little is known about the impairment of the CNS by T-cell lymphoma. The original paper entitled *Peripheral T-cell lymphomas involving central nervous system: a report from the Czech Lymphoma Study Group Registry NiHiL*, written by Mocikova et al., brings a retrospective description of patients with CNS involvement including incidence, survival differences, risk factors, and other data.

Across the world, survival rates after conventional chemotherapy regimens are poor for most subtypes of MTCL, and new therapies are needed. In contrast to B-cell lymphoma, no constant marker has been identified, which could be used as a therapy target (e.g., anti-CD20) with two exceptions present at least in part of T neoplasms: anaplastic lymphoma kinase (ALK) and CD30 antigen. The mentioned molecules are helpful in the diagnostic algorithm but, recently, also for the targeted therapy (e.g., brentuximab vedotin and crizotinib or alectinib).

The CD30 antibody–drug conjugate brentuximab vedotin (BV) has been applied as a single agent or in combination with frontline regimens. Encouraging results with a high response rate, good safety profile, and survival benefit were reported in newly diagnosed patients; on the other hand, in refractory/relapse (r/r) patients, although the overall reaction rate of BV was still impressive, the CR rate seemed unsatisfactory. Recently, a new immunotherapy technique, named chimeric antigen receptor (CAR) T-cell therapy, has been developed. CAR-T therapy targeting CD19 has been reported with exciting results in recurrent B-cell acute lymphoblastic leukemia (B-ALL) and in relapsing B-cell NHL. Based on the efficacy of the CAR-T therapy and CD30 expression exclusivity, the concept of an anti-CD30 CAR was designed. An overview of CAR-T therapy results (targeting CD30 and CD7 epitopes) and potential perspectives are summarized in a review written by Polgarova et al. entitled *Chimeric antigen receptor based cellular therapy for treatment of T-cell malignancies.*

ALK is a classical receptor tyrosine kinase belonging to the insulin receptor superfamily, which is strongly expressed in the nervous system during the embryonal phase. Activation of ALK stimulates many signaling pathways, including the RAS/RAF/MEK/ERK1/2, JAK/STAT, PI3K/AKT, and PLC-γ pathways. ALK aberration is found in several types of human cancers, which can be caused by different mechanisms. New research on the precise mechanism of ALK-mediated lymphomagenesis is published in the work entitled *NIPA (Nuclear Interaction Partner of ALK) is crucial for effective NPM-ALK mediated lymphomagenesis* by Kreutmair et al.

A better understanding of the pathogenetic mechanisms of T-cell lymphomagenesis and molecular alterations associated with specific lymphoma subtypes can be assessed using new biomarker approaches and can be potentially used for targeted therapy. In PTCL-NOS, for example, two molecular subgroups (PTCL-TBX21 and PTCL-GATA3) have been identified based on their gene expression profiling (GEP) resembling Th1 and Th2 cells, respectively. While the PTCL-GATA3 subgroup has greater genomic complexity, the PTCL-TBX21 subgroup has fewer copy number alterations and more frequent mutations in genes regulating DNA methylation. Although the designation of PTCL-NOS according to the molecular subgroups is not routinely incorporated into clinical diagnosis, an increasing number of epigenetic-modifying agents have created new opportunities for epigenetic therapies. Another approach for the identification of molecularly different diseases is presented by Veltri et al. in their original research paper entitled *Phosphoproteomic analysis reveals a different proteomic profile in pediatric patients with T-cell lymphoblastic lymphoma or T-cell acute lymphoblastic leukemia*. In this paper, the authors are able to establish an algorithm for the differentiation between two similar T-cell leukemias and give evidence that they are diverse diseases.

We would like to thank several authors who have contributed to this not easy but interesting and challenging topic. This Research Topic offers clinical experiences, future therapy perspectives, and also basic research on the subtle molecular nuances of specific T-cell lymphoproliferations. We also wish that all the readers of this Research Topic will be delighted with these published papers.

## Author contributions

The author confirms being the sole contributor of this work and has approved it for publication.

## Conflict of interest

The author declares that the research was conducted in the absence of any commercial or financial relationships that could be construed as a potential conflict of interest.

## Publisher’s note

All claims expressed in this article are solely those of the authors and do not necessarily represent those of their affiliated organizations, or those of the publisher, the editors and the reviewers. Any product that may be evaluated in this article, or claim that may be made by its manufacturer, is not guaranteed or endorsed by the publisher.

